# Impact of a Prenatal Nutrition Package with Balanced Energy Supplementation on Gestational Weight Gain in Amhara, Ethiopia

**DOI:** 10.1016/j.tjnut.2026.101653

**Published:** 2026-06-06

**Authors:** Yunhee Kang, Firehiwot Workneh, Kalkidan Yibeltal, Nebiyou Fasil, Estifanos Baye, Sitota Tsegaye, Workagegnhu Tarekegn Kidane, Yoseph Yemane Berhane, Fred Van Dyk, Krysten North, Grace J Chan, Sheila Isanaka, Rose L Molina, Amare Worku Tadesse, Nandita Perumal, Luke C Mullany, Alemayehu Worku, Blair J Wylie, Parul Christian, Yemane Berhane, Anne CC Lee

**Affiliations:** 1 Department of Food and Nutrition, Seoul National University, Seoul, South Korea; 2 Department of International Health, Johns Hopkins School of Public Health, Baltimore, MD, United States; 3 Department of Epidemiology and Biostatistics, Addis Continental Institute of Public Health, Addis Ababa, Ethiopia; 4 Department of Reproductive Health and Population, Addis Continental Institute of Public Health, Addis Ababa, Ethiopia; 5 Department of Global Health and Health Policy, Addis Continental Institute of Public Health, Addis Ababa, Ethiopia; 6 Department of Newborn Medicine, Brigham and Women’s Hospital, Boston, MA, United States; 7 Department of Nutrition and Behavioral Science, Addis Continental Institute of Public Health, Addis Ababa, Ethiopia; 8 Department of Pediatrics, Children’s Hospital of Philadelphia, Perelman School of Medicine, University of Pennsylvania, Philadelphia, PA, United States; 9 Department of Nutrition, Harvard T.H. Chan School of Public Health, Boston, MA, United States; 10 Beth Israel Deaconess Medical Center, Boston, MA, United States; 11 Department of Infectious Disease Epidemiology and International Health, London School of Hygiene and Tropical Medicine, London, United Kingdom; 12 Department of Epidemiology and Biostatistics, Arnold School of Public Health, University of South Carolina, Columbia, SC, United States; 13 Department of Pediatrics, Warren Alpert Medical School of Brown University, Providence, RI, United States; 14 Department of Public Health, Addis Continental Institute of Public Health, Addis Ababa, Ethiopia; 15 Department of Pediatrics, Harvard Medical School, Boston, MA, United States

**Keywords:** balanced energy and protein, gestational weight gain, pregnancy, Ethiopia, supplementation, undernutrition

## Abstract

**Background:**

Suboptimal gestational weight gain (GWG) is associated with adverse pregnancy outcomes; however, evidence on the impact of nutritional interventions on GWG in low-income settings remains limited.

**Objectives:**

This study aims to examine the effectiveness of an enhanced nutrition package (ENP), including a balanced energy protein (BEP) supplement, on GWG compared with routine care.

**Methods:**

We conducted a pragmatic cluster-randomized effectiveness study among pregnant women <24 wk of gestation in rural Amhara, Ethiopia. Twelve health centers were randomized to provide either ENP, including a daily BEP supplement for women with mid-upper arm circumference ≤23 cm, or routine care. GWG and GWG rate (kg/wk) were calculated from enrollment to late pregnancy weight measurement. Intention-to-treat (ITT), cluster-level analysis compared GWG outcomes between study arms. Primary analyses were restricted to women with observed third-trimester weights. Secondary dose–response analysis within the ENP arm examined associations between BEP consumption duration and GWG outcomes.

**Results:**

A total of 2392 women were enrolled and randomly assigned (*n* = 1210 ENP; *n* = 1189 routine care), with 2170 followed up until birth. In the ENP arm, 37% were eligible for BEP supplementation. ITT analysis showed no differences between arms in GWG rate [diff = –0.006 kg/wk; 95% confidence interval (CI): –0.042 kg/wk, 0.030 kg/wk] or observed GWG (diff = 0.009 kg; 95% CI: –0.687 kg, 0.706 kg). Among BEP-eligible women, GWG outcomes also did not differ between arms (ΔGWG rate = 0.006 kg/wk; 95% CI: –0.027 kg/wk, 0.030 kg/wk); observed ΔGWG = 0.201 kg (95% CI: –0.443 kg, 0.845 kg). In dose–response analysis within the ENP arm, women consuming BEP for 61–90 and >90 d had a higher GWG rate (61–90 d: 0.46 kg/wk, 95% CI: 0.003 kg/wk, 0.090 kg/wk); >90 d: 0.059 kg/wk; 95% CI: 0.018 kg/wk, 0.099 kg/wk) and observed GWG (61–90 d: 0.895 kg, 95% CI: 0.004 kg, 1.786 kg; >90 d: 1.206 kg, 95% CI: 0.331 kg, 2.081 kg) compared with those consuming BEP for <30 d.

**Conclusions:**

The ENP package, including BEP, did not improve GWG compared with routine care in this pragmatic study in the Ethiopian health system. Longer duration of prenatal BEP adherence (≥60 d) may benefit GWG and GWG rates among undernourished women.

This trial was registered as ISRCTN15116516.

## Introduction

Gestational weight gain (GWG) is widely used as an indicator of the adequacy of maternal nutrition during pregnancy. Greater weight gain in late pregnancy is associated with increased placental and birth weights [1]. The fetus and placenta account for approximately half of GWG, with the remaining attributed to blood volume expansion, extravascular fluid, breast mass, and maternal fat deposits [2]. Inadequate GWG is associated with adverse maternal and birth outcomes, including low birth weight (LBW), small-for-gestational age (SGA), and preterm birth [3]. In an analysis of data from low- and middle-income countries (LMIC), suboptimal GWG [weight gain below or above the Institute of Medicine (IOM) recommendations] has been associated with a wide range of adverse perinatal outcomes [4]. GWG is therefore a crucial indicator of pregnancy health and a key target for antenatal care (ANC), because it impacts outcomes and can be monitored and modified during pregnancy [2].

Balanced-energy protein supplementation to undernourished women during pregnancy may improve GWG and birth outcomes [5,6]. A recent meta-analysis based on 11 efficacy randomized controlled trials reported that prenatal balanced energy protein (BEP) was effective in increasing GWG percent adequacy by 6%, increasing estimated total GWG at delivery by 0.59 kg, and reducing the risk of severely inadequate and all inadequate GWG by 10% and 7%, respectively [6]. A 2021 systematic review revealed that maternal BEP supplementation reduced the prevalence of infants born SGA by 29%, LBW by 40%, and the rate of stillbirth by 61% in LMIC [3]. Imdad et al. [7] reported that balanced protein supplementation increased infant birth weight compared with control by 59.9 g (11 studies), with greater weight gain among babies of malnourished women. However, these meta-analyses had several limitations, such as heterogeneity in study design and comparison groups, in energy and macronutrient content of the BEP products tested and product formats, and in contexts [8].

The Enhancing Nutrition and Antenatal Infection Treatment (ENAT) trial (ISRCTN15116516) was an effectiveness study implemented in the Ethiopian health system that examined the independent and combined effects of an enhanced nutrition package (ENP) [iron-folic acid (IFA), iodized salt, intensive nutritional counseling, and for women with mid-upper arm circumference (MUAC) ≤23 cm, a daily micronutrient-fortified BEP] and an enhanced infection management package (EIMP) for genitourinary and parasitic infections on pregnancy outcomes (Supplemental Table 1) [9]. The primary outcomes of this pragmatic effectiveness study were infant birthweight and length. The intervention packages did not significantly impact the primary outcomes, though they reduced the prevalence of stillbirth [10].

The ENAT study provides a unique opportunity to evaluate the effectiveness of BEP supplementation on GWG in a real-world setting beyond the controlled efficacy settings. In this analysis, we examined the impact of the enhanced nutrition interventions in the ENAT study on the secondary outcomes of GWG rate (kg/wk) and observed GWG (kg). We examined the effectiveness of the intervention of the full nutrition package, as well as among the subset targeted for BEP supplementation (i.e., MUAC ≤23 cm). In secondary analysis, we further examine the relationship between BEP dose and GWG outcomes.

## Methods

The ENAT study was a pragmatic, open-label, 2 × 2 factorial, cluster-randomized effectiveness trial. The detailed protocol and primary impact results have been published elsewhere [10].

### Study setting

The study was conducted in 12 health centers located in West Gojjam and South Gondar, rural areas of northwestern Amhara, ∼2–6 h from the regional capital, Bahir Dar. Eligible health centers served a population of around 25,000, had an annual ANC volume exceeding 250 women, and a functioning laboratory. According to the National Household Consumption & Expenditure survey, 42.5% of households in the Amhara region were food insecure, which is higher than the 33.6% national average [11]. Pregnant women in the ENAT study area had limited dietary diversity due to restricted access to nutritious foods and cultural beliefs regarding food restrictions during pregnancy, with religious fasting being a common practice that reduced their food intake [12].

### Participants, enrollment, and randomization procedures

Pregnant women were eligible if they were ≤24 wk of gestation and lived within <2-h walking distance from health centers. Women were excluded from the study if they intended to relocate from the study area before delivery or had a nonviable fetus identified on the enrollment ultrasound. Study nurses obtained informed consent for participation from women before enrollment and randomization. Enrollment commenced in August 2020 and continued until December 2021.

Twelve health centers (each serving ∼25,000 population) were randomized with a 1:1 ratio to provide pregnant women with an ENP (*n* = 6 health centers) or routine care (*n* = 6 health centers). We used constrained randomization to achieve balance across key baseline indicators, including population size, ANC volume, number of births, and proximity to Bahir Dar. The study statistician (LCM) generated all potential random sequences for health center assignments, evaluated them based on predefined restriction criteria, and randomly selected 1 from the eligible sequences. Participants attending ANC were individually randomly assigned at enrollment to the EIMP or routine infection care. Each health center received a randomization list to EIMP or routine infection care in blocks of 4, 8, or 12, generated in *R* by the study statistician (LCM). Assignments were concealed in sequentially numbered opaque envelopes, opened by study nurses at enrollment [9].

### Study interventions

Interventions were delivered by health systems staff, reinforced by study nurses at monthly ANC visits, and were not masked to the study participants. The ENP arm included a monthly supply of adequately iodized salt (Waff Manufacturing, 30–40 ppm potassium iodate, 600 g bottles), enhanced IFA supply/counseling, and for women with MUAC ≤23 cm, a daily micronutrient-fortified BEP supplement (200 g daily sachet until delivery, 28 g protein, 784 kcal/d). The BEP consisted of a locally produced corn–soy flour blend (Super Cereal, Faffa Food Share Company) formulated to meet IOM-recommended levels for vitamins A, D, E, B2, B3, B6, B12, C, calcium, and phosphorus (Supplemental Table 1). In the routine nutrition care (i.e., not-ENP; control) arm, ANC services were provided per Ethiopian Ministry of Health guidelines, including weight monitoring and provision of daily IFA supplements. The EIMP consisted of screening and treatment for genitourinary tract infections (urinary tract infection, gonorrhea, and chlamydia) and deworming (mebendazole, followed by stool screening and treatment). In the routine infection care (i.e., not-EIMP; control) arm, women were treated for genitourinary tract infections synchronically, and received deworming once in pregnancy.

### Data collection

Trained research nurses collected data at enrollment and each monthly ANC visit, and at birth at the health center using tablets with the Survey Solutions (World Bank, v20.08, 2021) platform. Pregnancies were dated at enrollment with transabdominal obstetric ultrasonography at <24 wk using International Fetal and Newborn Growth Consortium for the 21st Century (INTERGROWTH-21st) equations [13]; detailed ultrasound methods are described elsewhere [9]. Maternal anthropometrics (weight and MUAC) were measured at every ANC visit until delivery. Research staff were trained and standardized in anthropometric measurements using INTERGROWTH-21st standard operating procedures. Maternal weight was measured using a high-quality digital scale (ADE M317600, Germany; precision 100 g), and height was measured with an adult stadiometer (Shorr Productions HeightLite). MUAC was measured to the nearest cm using insertion tapes. Each measurement was taken twice, with a third measurement conducted if the difference exceeded a minimum detectable threshold (0.5 kg weight, 0.1 cm MUAC). Daily equipment calibration checks were performed before measurements.

Adherence to BEP supplementation was assessed at each ANC study visit by BEP empty sachet count and maternal recall of consumption in the prior month. The BEP adherence duration was defined as the total number of days that BEP was consumed during the pregnancy. BEP consumption reported by maternal recall was highly and significantly correlated with empty sachet count (correlation coefficient = 0.95). Because of higher data availability, maternal recall of BEP adherence was used for this analysis.

### Outcome variables

GWG rate (kg/wk)/GWG (kg): GWG was defined as the difference in maternal weight (kg) between enrollment and the last measurement in the third trimester. GWG rate (kg/wk) was the rate of weight gain in pregnancy, defined as GWG (kg) divided by duration in time (wk) between enrollment and the last measurement. Negative values in GWG were considered implausible (*n* = 51).

GWG adequacy ratio (%): In sensitivity analyses, we estimated the GWG adequacy ratio (%), defined as the ratio of observed GWG over the recommended GWG based on the IOM BMI-specific guidelines, among participants who enrolled in the study in the first trimester [<13 wk of gestational age (GA)]. This outcome was not registered in ISRCTN15116516 and was added ex post. These analyses were restricted to a subset of participants in the trial who were enrolled at <13 wk because the first-trimester weight was used to approximate the prepregnancy BMI required to apply the IOM guidelines for expected weight gain, as follows:

*Expected GWG at the last observed weight measure = [(BMI specific expected first trimester weight ÷ 13.86 wk) × (13.86 wk − GA at first observed or imputed weight measure)] + [(GA at last weight measure − 13.86 wk) × BMI specific recommended mean rate of GWG in the second and third trimesters]* [4]

The IOM BMI-specific expected weight gain for the number of weeks since the observed first-trimester weight until the end of the first trimester (13.86 wk: 13 wk and 6 d) was 2 kg for BMI <18.5 or 18.5 ≤ BMI < 25; 1 kg for 25.0 ≤ BMI < 30; and 0.5 kg for BMI ≥30. The recommended BMI-specific weight gain in the second and third trimesters is 0.51 kg/wk for BMI <18.5; 0.42 kg/wk for BMI <18.5 to <24.9; 0.28 kg/wk for BMI <25.0 to <30; and 0.22 kg/wk for BMI ≥30.

In sensitivity analysis, we imputed weights of participants who were randomly assigned but did not have weight measures at the third trimester (>28 wk) to calculate the *GWG rate and GWG.* First, we prepared a longitudinal data set of available weight at each visit per individual. To reduce the influence of extreme values, observations outside the 1st and 99th percentiles of GA were excluded. GA was centered at 36 wk (the mean age at the last ANC visit). We fitted a 3-level linear mixed-effect model with maternal weight as the outcome. Fixed effects included a quadratic term of GA at each weight measurement, baseline weight, GA at enrollment, maternal age, BMI, education, and occupation. Random intercepts were specified at the health center and individual levels, with a random slope for GA at the individual level. Fixed-effect predictions were computed at 36 wk of gestation, where the centered GA term equals zero. These fixed effects were combined with the estimated random intercept at the facility and individual levels to generate personalized predictions of maternal weight at 36 wk. For participants with only 1 weight record, the population-level fixed-effect prediction was substituted. After this process, we had 507 additional imputations; of these, we excluded 91 because they indicated negative weight change.

### Statistical analysis

The ENAT study’s statistical analytic plan was published online [9]. We hypothesized that the main intervention effects on GWG were primarily due to the nutrition package and thus assessed the effects of the ENP package (ENP or ENP + EIMP) compared with routine nutrition care (EIMP or neither package). We followed ITT principles and used factorial analysis to determine marginal ENP intervention effects in the absence of interaction. The primary analysis was a complete case analysis, including participants with both enrollment and third-trimester weights. Study participants with no weight measured during the third trimester were excluded from the primary analysis. In sensitivity analysis, we included imputed third-trimester weights for those missing outcomes (above). All outcomes and covariates were presented as mean (SD) or *n* (%) at enrollment and the third-trimester visit. For anthropometric measures, the mean of 2 measures was calculated, and for mothers with 3 measurements, the median value was used.

We performed descriptive analyses of variables at the individual and cluster levels to assess randomization balance. Comparability between the ENP and routine care groups was assessed with a Student *t*-test or a χ^2^ test, as appropriate. Cluster-level analysis was used to estimate the intervention impact of ENP compared with routine care on observed GWG and GWG rate, adjusting for enrollment weight and GA, and imbalanced baseline covariates. For the analysis of total GWG, the effects were adjusted for the duration of follow-up between enrollment and third-trimester visits. We also conducted subgroup analysis by enrollment nutritional status: MUAC >23.0 cm compared with ≤23 cm and enrollment BMI ≥18.5 compared with <18.5 kg/m^2^. The intervention effects on the GWG adequacy ratio (%) were estimated only among women who enrolled in the first trimester.

As a secondary analysis, we conducted a per-protocol analysis, restricting the intervention sample to women who consumed ≥60 d of BEP. To improve comparability between groups, we limited the routine care group for this analysis to those consuming ≥60 d of IFA supplementation. We also conducted a secondary observational dose–response analysis among women in the intervention arm to determine the association of the intensity of BEP consumption [≤30 d (reference), 31–60 d, 61–90 d, and ≥91 d] and GWG outcomes using linear regression analysis adjusting for potential confounders (maternal age, weight, enrollment BMI, education, occupation, and GA at enrollment). SEs were adjusted for clustering by health center using the *vce (cluster)* function in STATA.

Research ethics approval: the ENAT protocol was approved at Addis Continental Institute of Public Health (001-A1-2019) and Mass General Brigham Institutional Review Board (2018P002479). The Amhara Public Health Institute granted local permission to conduct the study. The study was prospectively registered at ISRCTN (ISRCTN15116516).

## Results

**FIGURE 1:**
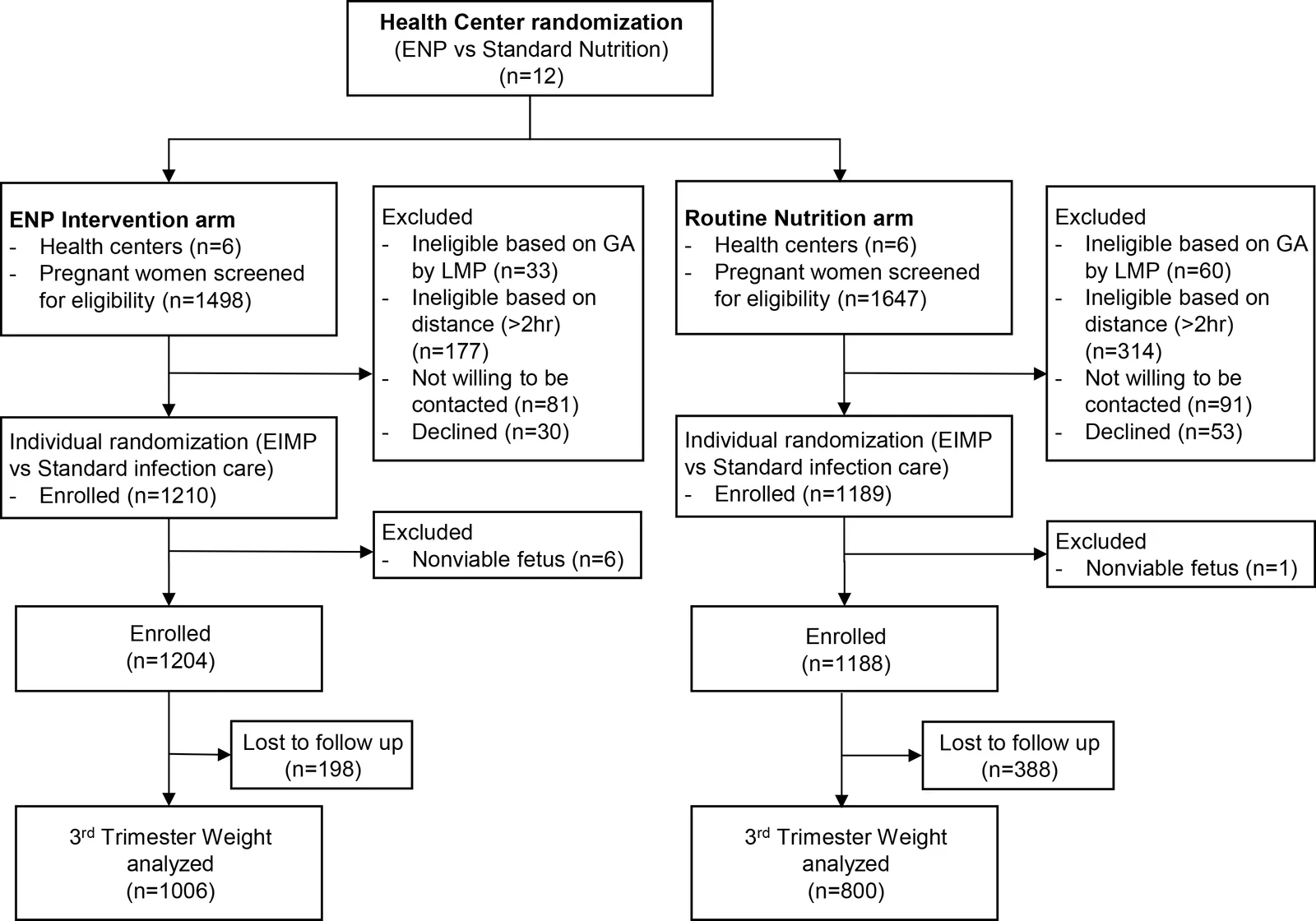
Study flow. ENP, enhanced nutrition package; EIMP, enhanced infection management package; GA, gestational age; LMP, last menstrual period.

The CONSORT flowchart is shown in Figure 1. Between 3 August, 2020 and 10 December 10, 2021, we screened 3145 women for eligibility and enrolled 2399 pregnant women, of whom 1204 were enrolled at ENP health centers and 1188 at routine nutrition care health centers. A total of 2170 pregnancies were followed up for birth outcomes (1114 ENP and 1056 routine care). A total of 1806 pregnant women had a follow-up third-trimester ANC visit with a weight measurement. Follow-up rates and duration were higher in the ENP arm [1006 third-trimester visits; mean follow-up 19.5 wk (SD 5.9)] compared with routine care [800 third-trimester visits; mean follow-up 18.7 wk (SD 6.0)]. Overall, compared with mothers with third-trimester visits, mothers who were without weights (*n* = 586) were more likely to be older, engaged in agriculture, and of a lower education level (Supplementary Table 2).

### Baseline characteristics

Maternal age was similar across study arms. Women in the ENP arm tended to be less educated, with a higher proportion having no formal education or only primary-level education than the routine care arm (Table 1). Engagement in agricultural occupations was higher among ENP mothers (55.2%) than routine care mothers (41.5%). Women in the ENP arm had a lower baseline weight [51.2 (SD 6.6) kg compared with 51.9 (SD 6.7) kg] and a higher proportion with low BMI (<18.5 kg/m^2^; 19.7% compared with 13.4%) compared with women in the routine care arm.

**TABLE 1:** Baseline characteristics of study participants, by ENAT Nutrition allocation arm (*n* = 1806)

Characteristics	ENP 6 clusters (health centers) (*n* = 1006 women)	Routine care, 6 clusters (health centers) (*n* = 800 women)
Gestational age at enrollment, wk, mean (SD)	16.5 (5.4)	16.4 (5.4)
Age (y), mean (SD)	25.9 (5.6)	26.2 (5.3)
Parity		
Nulliparous, *n* (%)	312 (31.0)	258 (32.3)
Education level, *n* (%)		
No education	472 (47.1)	339 (42.3)
Primary	310 (31.0)	314 (28.3)
Secondary or higher	219 (21.9)	303 (29.4)
Missing, *n*	5	2
Occupation, *n* (%)		
No formal occupation	259 (25.9)	275 (34.4)
Agriculture	553 (55.2)	332 (41.5)
Waged job	190 (19.0)	193 (24.1)
Missing, n	4	0
Weight, kg, mean (SD)	51.2 (6.6)	51.9 (6.7)
BMI (kg/m^2^) at enrollment, mean (SD)	20.5 (2.4)	20.8 (2.4)
BMI category (kg/m^2^), *n* (%)		
<18.5	198 (19.7)	107 (13.4)
18.5–24.9	765 (76.1)	653 (81.6)
≥25.0	42 (4.2)	40 (5.0)
Missing data, no	1	0
MUAC (cm) at enrollment, mean (SD)	24.1 (2.2)	23.9 (1.9)
MUAC ≤23 cm, %	37.2	34.5
Duration from enrollment to the last ANC visit, mean (SD)	19.6 (5.9)	18.7 (6.0)

	*n* = 969	*n* = 750

Duration (wk) from enrollment to pregnancy outcomes, mean (SD)	23.3 (5.4)	23.4 (5.6)

Abbreviations: ANC, antenatal care; ENAT, enhancing nutrition and antenatal infection treatment; ENP, enhanced nutrition package; MUAC, mid-upper arm circumference.

### Effect of full ENP package compared with routine care on GWG

In the ENP arm, the GWG rate and observed GWG were 0.29 (SD 0.14) kg/wk and 5.52 (SD 3.10) kg, respectively, whereas in the routine care arm, the GWG rate was 0.30 (SD 0.17) kg/wk and the observed GWG was 5.57 (SD 3.41) kg. There was no significant difference in the GWG rate {–0.006 [95% confidence interval (CI): –0.042, 0.030] kg/wk} or observed GWG [0.009 (95% CI: –0.60, 0.71) kg] between the ENP and routine care group based on cluster-level analysis of 12 clusters (Table 2A). In sensitivity analysis using the full enrolled cohort, including imputed missing third-trimester weights, there was no effect of ENP on GWG rate (Supplemental Table 3A) [–0.005 (95% CI: –0.037, 0.026) kg/wk] or observed GWG [0.027 (95% CI: –0.67, 0.73) kg]

**TABLE 2:** Effectiveness of ENAT enhanced nutrition package on gestational weight gain (kg) and weight gain rate (kg/wk) in full cohort and among subgroups (*n* = 1806)

Outcomes	Total, mean (SD)	ENP, mean (SD)	Routine care mean (SD)	Unadjusted mean difference^1^ (95% CI)	Adjusted mean difference (95% CI)^2^
A. Total ENAT cohort	*n* = 1806	*n* = 1006	*n* = 800		

GWG rate (kg/wk)	0.293 (0.156)	0.286 (0.142)	0.302 (0.172)	–0.007 (–0.046, 0.033)	–0.006 (–0.0042, 0.030)
Observed GWG (kg)	5.542 (3.243)	5.517(3.103)	5.574 (3.411)	0.186 (–0.638, 1.011)	0.009 (–0.687, 0.706)

B. Cohort with MUAC ≤23 cm^3^ (targeted for BEP)	*n* = 644	*n* = 374	*n* = 270		

GWG rate (kg/wk)	0.296 (0.147)	0.294 (0.142)	0.297 (0.153)	0.008 (–0.029, 0.046)	0.006 (–0.027, 0.039)
Observed GWG (kg)	5.651 (3.126)	5.708 (3157)	5.572 (3.087)	0.302 (–0.403, 1.007)	0.201 (–0.443, 0.845)

C. Cohort with MUAC ≤23 cm and higher BEP adherence (>60 d)^3^^,^^4^	*n* = 268	*n* = 169	*n* = 99		

GWG rate (kg/wk)	0.307 (0.133)	0.304 (0.127)	0.311 (0.143)	–0.001 (–0.038, 0.035)	0.002 (–0.031, 0.037)
Observed GWG (kg)	5.974 (3.002)	6.123 (3.021)	5.720 (2.966)	0.355 (–0.460, 1.170)	0.111 (–0.672, 0.893)

Abbreviations: BEP, balanced energy-protein; CI, confidence interval; ENAT, Enhancing Nutrition and Antenatal Infection Treatment; ENP, enhanced nutrition package; GWG, gestational weight gain; MUAC, mid-upper arm circumference.

1 Cluster analysis based on 12 health centers was used to derive the mean difference (95% CI).

2 Accounted for maternal age, weight, BMI, education, occupation, gestational age weeks at enrollment, and duration from enrollment and third trimester (only for observed GWG), accounting for health center location.

3 MUAC ≤23 cm group was targeted with BEP supplementation in the ENP arm.

4 Comparison of higher adherence >60 d BEP in ENP intervention arm, to women with >60 d of iron and folic acid adherence in routine care arm.

### Effect of ENP compared with routine care on GWG adequacy ratio

Among the 451 women with first-trimester weights, the GWG adequacy ratio was calculated (Supplemental Table 4). There were no significant effects on GWG rate [–0.000 (95% CI: –0.001, 0.001)], observed GWG [–0.000 (95% CI: –0.002, 0.002) kg], kg/wk, or GWG adequacy [7.8% (95% CI: –2.9%, 18.6%)].

### ENP impact among a subset of women with MUAC <23 cm targeted for BEP

Overall, 34.5% of women in the routine care arm and 37.2% of women in the ENP arm ever had a MUAC of ≤23 cm at any point during pregnancy. The mean duration of BEP consumption in the ENP arm was 71.4 (SD 50.0) d. The GWG rate and observed GWG were 0.29 (SD 0.14) kg/wk and 5.71 (SD 3.16) kg in the ENP arm, and in the routine care arm, the GWG rate was 0.30 (SD 0.153) kg/wk, and the observed GWG was 5.57 (SD 3.09) kg. There was no significant difference in GWG rate [0.006 (95% CI: –0.027, 0.039) kg/wk] or observed GWG [0.201 (95% CI: –0.443, 0.845) kg] among mothers with MUAC ≤23 cm between the ENP and routine care arms (Table 2B). In sensitivity analysis, including the full cohort, where third-trimester weights were imputed for those with missing third-trimester weights, no significant ENP effects were seen on GWG rate/observed GWG (Supplemental Table 3B).

### ENP impact among a subset of women with MUAC <23 cm and >60 d BEP compliance

Among women who consumed BEP for >60 d (*n* = 169), the mean GWG rate was 0.304 (0.127) kg/wk, compared with 0.311 (SD 0.143) kg/wk among those in the routine care arm not receiving BEP but consuming IFA for >60 d. The difference was not statistically significant [mean difference: 0.002 kg/wk (95% CI: –0.031 kg/wk, 0.037 kg/wk)]. The mean observed GWG was higher among women consuming BEP for >60 d compared with those in routine care consuming IFA for >60 d, but there was no significant difference in observed GWG [0.111 kg (–0.672, 0.893)] (Table 2C).

### Subgroup analysis

In subgroup analysis stratified by maternal baseline BMI status (<18.5 compared with ≥18.5 kg/m^2^), there were no significant effects on GWG rate or observed GWG (Supplemental Table 5). Similarly, no significant effects were observed after imputing maternal third-trimester weight (Supplemental Table 3C). Likewise, there were no significant intervention effects among women with MUAC >23 cm (Supplemental Table 5).

### Dose–response analysis within intervention arm: duration of BEP consumption and GWG outcomes

**FIGURE 2:**
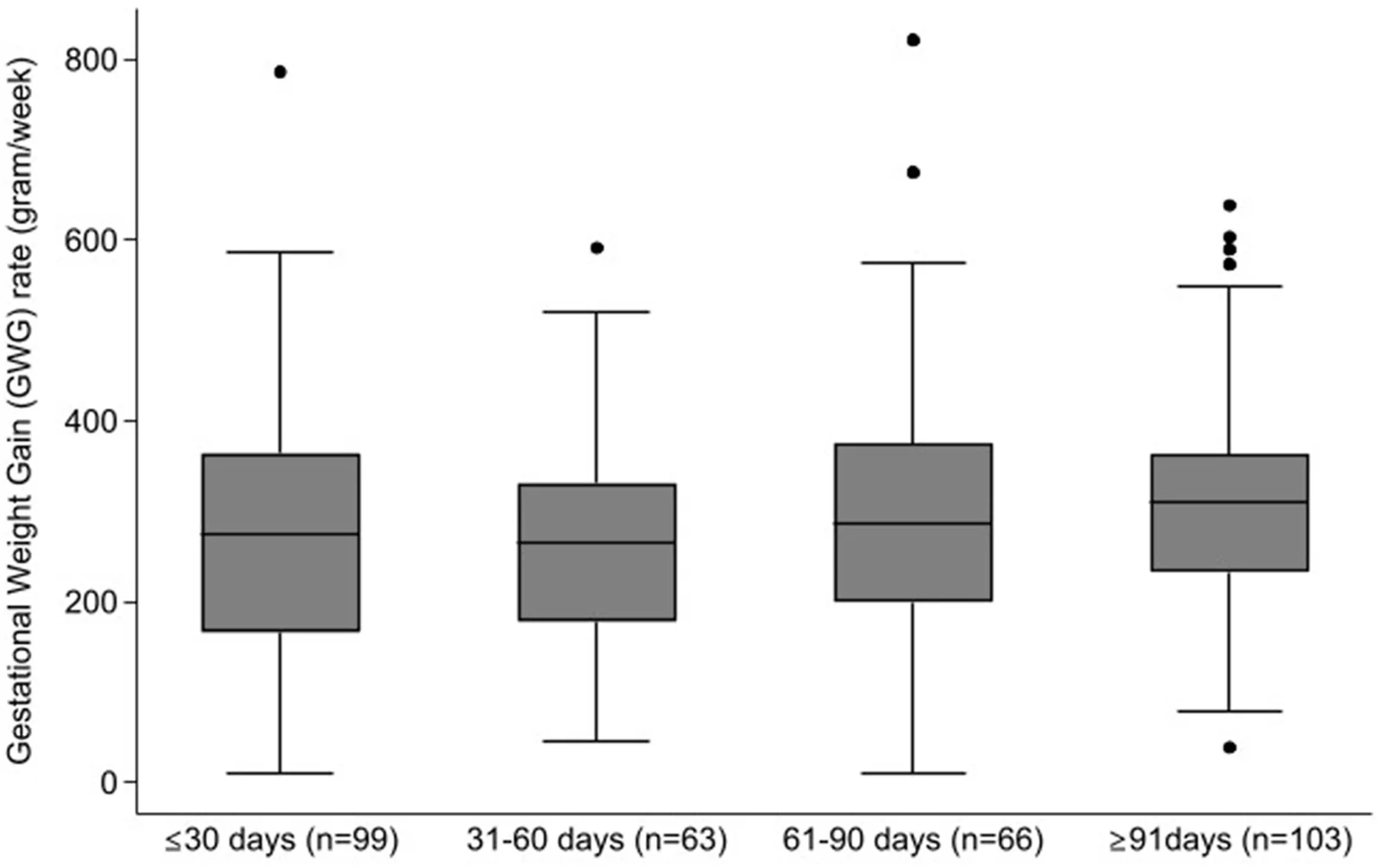
Gestational weight gain (GWG) rate by intensity of BEP consumption among ENP group (*n* = 331). BEP, balanced energy protein; ENP, enhanced nutrition package.

Figure 2 illustrates the association between the duration of BEP consumption and GWG outcomes within the ENP arm. Overall, 34.3%, 19.1%, 18.3%, and 28.3% of women consumed BEP for <30, 31–60, 61–90, and ≥91 d, respectively. In observational analyses, the GWG rate and observed GWG increased with the longer durations of BEP consumption (Table 3, Figure 2). Adjusting for potential confounders that could bias supplement compliance, women who consumed BEP for 61–90 d or ≥91 d had 0.895 kg (95% CI: 0.004 kg, 1.786 kg) and 1.206 kg (95% CI: 0.331 kg, 2.081 kg) higher GWG, respectively, than women who consumed BEP for <30 days. Women who consumed BEP for ≥91 days had a 0.059 kg/wk (95% CI: 0.018 kg/wk, 0.099 kg/wk) higher GWG rate compared with those consuming BEP for ≤30 d (Table 3).

**TABLE 3:** Observational analysis in the ENP arm

	≤30 d in BEP (active arm, meeting the ≤23 cm MUAC) (*n* = 99)	31–60 d in BEP (active arm) (*n* = 63)	61–90 d in BEP (active arm) (*n* = 66)	≥91 d in BEP (active arm) (*n* = 103)
GWG rate (kg/wk), mean (SD)	0.274 (0.147)	0.273 (0.128)	0.298 (0.139)	0.307 (0.119)
Unadjusted mean difference in GWG rate (kg/wk) (95% CI)^1^	Ref	0.006 (–0.054, 0.067)	0.037 (–0.010, 0.084)	0.046 (0.021, 0.071)
Adjusted mean difference in GWG rate (kg/wk) (95% CI)^2^	Ref	0.021 (–0.032, 0.074)	0.046 (0.003, 0.090)∗	0.059 (0.018, 0.099)∗
Observed GWG (kg), mean (SD)	5.293 (3.204)	4.890 (2.783)	5.594 (3.048)	6.462 (2.970)
Unadjusted mean difference in observed GWG (kg) (95% CI)^1^	Ref	–0.025 (–1.592, 1.103)	0.058 (–0.084, 1.997)	1.408 (0.039, 2.430)∗
Adjusted mean difference in observed GWG (kg) (95% CI)^2^	Ref	0.485 (–0.446, 1.416)	0.895 (0.004, 1.786)∗	1.206 (0.331, 2.081)∗

BEP dose response: observed gestational weight gain (GWG, kg) and weight gain rate (kg/wk) by intensity of BEP consumption among women with MUAC ≤23.0 cm (*n* = 331).

Abbreviations: BEP, balanced energy-protein; CI, confidence interval; ENP, enhanced nutrition package; GWG, gestational weight gain; MUAC, mid-upper arm circumference.

1 Linear regression models were used to derive *P* value.

2 Accounted for maternal age, weight, BMI, education, occupation, and gestational age weeks at enrollment, and the duration (wk) between enrollment and third trimester; accounted for health center clustering.

∗ *P* < 0.05.

## Discussion

This pragmatic effectiveness study implemented within the Ethiopian health system examined the effect of an ENP, including targeted BEP supplementation to undernourished women, on weekly GWG rate and observed GWG in pregnancy. In the ITT analysis, we found no significant differences in the GWG outcomes between mothers who received the enhanced nutrition intervention package and those who received routine care (IFA). Approximately 1 in 3 women was malnourished (MUAC <23 cm) and targeted for BEP supplementation in the intervention group. We also found no intervention effect in the subgroup of women eligible for BEP, which may have been due to relatively low adherence. In per-protocol analysis limited to mothers with higher BEP adherence (≥60 d), there was a trend of higher GWG (but not GWG rate) in the supplemented group, but this was not statistically significant. Among women in the intervention arm, we found that consumption of 60–90 d or ≥91 d of BEP was associated with a higher weekly GWG rate and observed GWG, compared with consumption of fewer than 30 d of BEP, although this observational analysis should be interpreted with caution, given potential residual confounding.

We did not observe an impact of the ENP on GWG, which could be due to relatively low adherence to the BEP supplement. The adherence to BEP supplementation among participants in the study was moderate, with a mean total consumption of 71.4 d/sachets during pregnancy, amounting to 52% of eligible days. Delivery of the BEP was at the health center, and travel during the study period was limited because of both the COVID-19 pandemic and local civil unrest. Within the ENP arm, in the observational dose–response analysis, increasing BEP supplementation duration (60–90 or >90 d) was significantly associated with greater GWG and GWG rate compared with supplementation for <30 d. In a controlled efficacy setting such as the MIcronutriments pour la SAnté de la Mère et de l'Enfant (MISAME) trial in Burkina Faso, the observed BEP supplement adherence rate was 83.1% [14]. Wang et al.’s [6] meta-analysis demonstrated the benefit of BEP supplementation starting before 20 wk of gestation, which leads to longer supplementation duration. In our study, the median GA at which supplementation started was 16.7 wk. Apart from behavioral compliance, the limited effectiveness may potentially be due to impaired absorption or enteric dysfunction among undernourished women [15].

Several other factors may explain the lack of impact of the ENP intervention on GWG. There was an imbalance in enrollment nutritional status between the study clusters/intervention arms, with the nutrition arm being more disadvantaged at baseline. In the ENP intervention clusters, the proportion of underweight participants (BMI <18.5) at enrollment was higher than in the routine care group (19.7% compared with 13.4%), and the proportion of mothers with MUAC ≤23 cm was higher (37.2% compared with 34.5%). Although we adjusted for baseline maternal weight in our adjusted models, it is possible that we were not able to adequately account for the baseline imbalance. Because of the small cluster number (*n* = 12 health centers), we had lower statistical power to detect our program’s effects. Religious fasting is also another potential explanation for the lack of impact, as Orthodox Christian doctrine considers >200 d/y as fasting days, when animal products and dairy are restricted, and meals are delayed until 15:00 [12]. Although pregnant women are formally exempt, our formative research indicated that many continued to fast during pregnancy because eating was perceived as irreligious and potentially displeasing to God [12]. Also, although the BEP was intended to supplement regular meals, it is plausible that the BEP supplement may have displaced regular food or nutrient intake. We conducted extensive counseling regarding the supplementation prior to the trial and are currently analyzing to assess food displacement. Reductions in household meal frequency and portion size, as well as limited availability of animal-source foods within the community, may constrain dietary intake and thereby limit the ability to achieve adequate GWG. Finally, environmental and biological factors such as frequent infections (e.g., geohelminths) may reduce appetite or increase metabolic demands, as well as environmental enteric dysfunction [16,17] that may impact nutrient absorption. Together, these factors may have limited dietary intake and the effectiveness of nutritional interventions on GWG. Together, these environmental, biological, and social constraints, beyond supplementation delivery alone, may play an important role in modifying GWG outcomes.

Although several efficacy trials have previously examined the effect of prenatal BEP supplementation on GWG outcomes in LMIC, few have examined real-world implementation outcomes in health systems. A recent meta-analysis based on BEP effects on GWG showed that balanced energy and protein supplements increased GWG percent adequacy by 6% and total GWG at delivery by 0.59 kg, and reduced risk of inadequate GWG by 10% [6]. Unlike the controlled efficacy trials, our study employed a pragmatic effectiveness design by integrating BEP supplementation into routine ANC within the existing health system. Our study provided valuable real-world evidence on maternal compliance with BEP supplementation and the extent to which it contributes to improving GWG among pregnant women in noncontrolled service delivery settings. We also observed a tendency that may suggest a potential threshold benefit, although this requires further confirmation in future studies. The IOM provides recommended GWG ranges based on prepregnancy BMI categories, recommending 11.5 kg for normal weight and 12.5 kg for underweight women [2]. These recommendations were derived from data from mothers in high-income countries. In our study, the imputed GWG from the first trimester to delivery was 8.31 kg, still lower than the minimum recommendations (Supplemental Table 5). However, our estimated GWG at delivery is consistent with the range reported in a recent meta-analysis of 10 BEP effect studies in LMICs, which estimated a mean GWG of 9.1 kg (range: 7.5–12.2 kg) [6]. In 2 sub-Saharan countries, the estimated mean weight gain was 6.64 kg among women in sub-Saharan Africa, indicating <60% of the IOM-recommended GWG range [18].

### Strengths and limitations

This study has several strengths and limitations. This study was conducted as a cluster-randomized effectiveness trial; all intervention delivery was performed within the health system, and all measurements were performed with rigorous quality and quality control standards. We had high-quality birthweight and GA data. There are a few limitations. First, it was not feasible to directly measure GWG across the entire pregnancy. One in 5 women was enrolled in the first trimester, and the final weight measurement was ∼36 wk of gestation. In a sensitivity analysis, we imputed weight at delivery among women whose weight was measured in both the first and third trimesters.

Loss to follow-up was imbalanced between the ENP and routine care groups. The nutritional supplements were a substantial demand driver for return to follow-up ANC during the COVID pandemic, and rates of third-trimester follow-up were lower in the routine care arm. To address potential bias, we applied an imputation approach in sensitivity analysis to estimate missing third-trimester weight outcomes, thereby improving the plausibility of the conditionally missing-at-random assumption. Although the loss to follow-up was imbalanced by study arm, characteristics of those lost to follow-up were similar across study arms (ENP compared with routine care) with respect to age, education, and nutritional status. We also adjusted for potential confounders in our effect estimates to reduce bias.

In conclusion, in this pragmatic effectiveness study, the ENP package, including BEP, did not improve observed GWG or GWG rate in rural Ethiopia. The lack of effectiveness may have been due to suboptimal BEP supplement adherence and baseline imbalance between study arms, including a greater baseline risk of undernutrition in the ENP intervention arm. Longer duration of BEP consumption during pregnancy (e.g., a minimum of 60 d) was associated with higher weekly GWG rates and observed GWG among undernourished women. Future research should address implementation barriers to BEP compliance in this and other settings.

## Author contributions

The authors’ responsibilities were as follows – ACL, YB, AW, LCM, PC, FW, GJC, SI, RLM, AWT, BJW: contributed to the study conceptualization and design; FW, KY, ST, WTK, AW, EB: contributed to the intervention package development and delivery, including clinical protocols and laboratory analyses; FW, KY, ST, WTK, EB: provided project administration, management, and supervision; FW, YK, KY, KN, NF, ST, YYB, FVD, EB: contributed to data acquisition and curation; YK: conducted statistical analysis with inputs from PC, NP, and ACL, and wrote the original manuscript draft; and all authors: read and approved the final version.

## Data availability

Data described in the manuscript, codebook, and analytic code will be made available upon request, pending application and approval.

## Funding

This work was funded by the Bill and Melinda Gates Foundation (OPP1184363/INV-010109, INV-045894).

## Declaration of generative AI and AI-assisted technologies in the writing process

The author(s) declare that no generative AI or AI-assisted technologies were used in the writing of this manuscript.

## Conflict of interest

ACL received support from the Eunice Kennedy Shriver National Institute of Child Health and Human Development (5K23HD091390), World Health Organization, and Johns Hopkins University. All other authors report no conflicts of interest.
