# Supplementary material for: Impact of a Prenatal Nutrition Package with Balanced Energy Supplementation on Gestational Weight Gain in Amhara, Ethiopia
Source: J Nutr. 2026 Jun 6;156(8):101653. doi: 10.1016/j.tjnut.2026.101653 (PMC13494173; doi:10.1016/j.tjnut.2026.101653)
Supplement: Supplementary file 1 [file mmc1.docx]

**Impact of a Prenatal Nutrition Package with Balanced Energy Supplementation on Gestational Weight Gain in Amhara, Ethiopia (First Author: Yunhee Kang)**

**Supplementary Table 1. Balanced Energy Protein Supplement Composition (FAFFA Super Cereal)**

**Dose: 200 g sachet once daily to supplement regular meals**

| Nutrient | Content (per 200g) | Unit | Recommended Ranges in Pregnancy | |
| --- | --- | --- | --- | --- |
| Macronutrient |  |  | BMGF Expert Consultation^Δ^ | |
| Energy (Kcal) | 760 | kcal | 250-500 kcal, in high-risk population portion size can be doubled | |
| Protein | 28 | g | 16 g (50% of additional protein requirement in 3^rd^ trimester) | |
| Fat | 12 | % | 10-60% of energy | |
| Micronutrient |  |  | IOM  Minimum/Target (EAR)* | IOM  Maximum (RDA)* |
| Vitamin A | 2076 | mcg RE | 550 | 770 |
| Vitamin D3 | 22.08 | µg | 10 | 15 |
| Vitamin E TE | 16.6 | mg | 16 | 19 |
| Vitamin K1 | 60 | µg | 72 | 90 |
| Vitamin B1 | 0.4 | mg | 1.2 | 1.4 |
| Vitamin B2 | 2.8 | mg | 1.3 | 1.6 |
| Niacin B3 | 16 | mg | 14 | 18 |
| Vitamin B6 | 2 | mg | 1.7 | 2 |
| Folic Acid, B9 | 220 | µg | 400 | 600 |
| Vitamin B12 | 4 | µg | 2.4 | 2.8 |
| Vitamin C | 180 | mg | 100 | 120 |
| Iron | 8 | mg | 22 | 27 |
| Zinc | 10 | mg | 15 | 20 |
| Iodine | 80 | µg | 209 | 290 |
| Copper | 0 | mg | 1.0 | 1.3 |
| Selenium | 0 | µg | 60 | 70 |
| Calcium | 724 | mg | 500 | 1000 |
| Phosphorus | 560 | mg | 300 | 700 |

BMFG, Bill & Melinda Gates Foundation; EAR, estimated average requirement; IOM, Institutes of Medicine. MUAC=mid-upper arm circumference; RDA, recommended dietary allowance.

Δ Bill and Melinda Gates Foundation Expert Consultation on Framework and Specifications for Nutritional Composition of a Food Supplement for Pregnant and Lactating Women in Undernourished and Low-Income Settings. April 2017

*IOM. Dietary Reference Intakes: The Essential Guide to Nutrient Requirements. The National Academies Press, Washington, D.C., 2006. EAR was used for minimum value and RDA for maximum value.

**Supplementary Table 2. Characteristics of Women Lost to Follow-up**

| **Characteristic** | **Lost to follow up**  **ENP**  **n=198** | **Lost to follow up**  **Routine care**  **n=388** |
| --- | --- | --- |
| **Maternal age**  **Mean (sd)** | 27.1 (5.9) | 27.1 (5.8) |
| **Maternal education, n(%)** |  |  |
| No education | 118 (62.1) | 2224 (59.0) |
| Primary | 47 (24.7) | 88 (23.2) |
| Secondary or higher | 25 (13.2) | 68 (17.9) |
| **Maternal occupation, n(%)** |  |  |
| Informal | 33 (17.4) | 103 (27.1) |
| Agriculture | 120 (63.2) | 199 (52.4) |
| Waged occupation | 37 (19.5) | 78 (20.5) |
| **Maternal weight at enrollment**  **Mean (sd)** | 51.6 (6.2) | 51.6 (7.3) |
| **Maternal BMI at enrollment, n(%)** | 20.7 (2.2) | 20.7 (2.5) |
| <18.5 | 33 (17.6) | 63 (16.8) |
| 18.5-24.9 | 148 (78.7) | 290 (77.3) |
| ≥25.0 | 7 (3.7) | 22 (5.9) |
| **Maternal MUAC at enrollment**  **Mean (sd)** | 24.2 (2.1) | 23.7 (2.1) |
| Maternal MUAC < 23 cm | 45 (23.8) | 116 (33.2) |

ENAT, Enhancing Nutrition and Antenatal Infection Treatment; ENP, Enhanced Nutrition Package; ANC, antenatal care; MUAC, mid-upper arm circumference; BMI, body mass index

**Supplemental Table 3. Effectiveness of ENAT Enhanced nutrition package on gestational weight gain (kg) and weight gain rate (kg/wk) in full cohort (imputation of missing weights) and among subgroups (n=2274)**

| Outcomes | Total  mean (SD) | ENP  mean (SD) | Routine care mean (SD) | Unadjusted mean difference^1^ (95%CI) | Adjusted mean difference  (95%CI)^2^ |
| --- | --- | --- | --- | --- | --- |
| **A. Total ENAT Cohort** | n=2274 | n=1143 | n=1131 |  |  |
| GWG rate (kg/week), | 0.289 (0.139) | 0.284 (0.132) | 0.295 (0.145) | -0.006 (-0.016, 0.016) | -0.005 (-0.037, 0.026) |
| Observed GWG (kg) | 5.573 (3.000) | 5.553 (2.962) | 5.592 (3.000) | 0.068 (-0.557, 0.693) | 0.027 (-0.670, 0.725) |
| **B. Cohort with MUAC≤23 cm*** (Targeted for BEP) | n=795 | n=413 | n=382 |  |  |
| GWG rate (kg/week) | 0.293 (0.131) | 0.292 (0.134) | 0.294 (0.128) | 0.004 (-0.028, 0.036) | -0.001 (-0.028, 0.026) |
| Observed GWG (kg) | 5.681 (2.904) | 5.721 (3.045) | 5.638 (2.747) | 0.118 (-0.434, 0.671) | 0.227 (-0.407, 0.860) |
| **C. Cohort with MUAC≤23 cm and higher BEP adherence (>60 days)* in ENP** | n=293 | n=171 | n=122 |  |  |
| GWG rate (kg/week) | 0.305 (0.127) | 0.303 (0.125) | 0.308 (0.130) | -0.001 (-0.034, 0.034) | 0.000 (-0.031, 0.031) |
| Observed GWG (kg) | 5.880 (2.912) | 6.100 (3.012) | 5.575 (2.749) | 0.488 (-0.209, 1.185) | 0.129 (-0.669, 0.927) |

ENAT, Enhancing Nutrition and Antenatal Infection Treatment; ENP, Enhanced Nutrition Package; BEP, Balanced energy-protein; GWG, gestational weight gain; MUAC, mid-upper arm circumference

^1^ Cluster analysis based on 12 HC was used to derive β (95%CI)

^2^ Accounted for maternal age, weight, BMI, education, occupation, GA wks at enrollment, and duration from enrollment and 3rd trimester (only for observed GWG), accounting for health center location

* MUAC **≤**23 cm group was targeted with Balanced Energy Protein Supplementation in the Enhanced Nutrition Package Arm

**Supplementary Table 4. Enhanced nutrition package effects on Adequacy rate of gestational weight gain (GWG), GWG rate (kg/week), and observed GWG (kg) among women who enrolled in the 1^st^ trimester and singleton liveborn only (n=451)**

|  | Total  Mean (SD) | ENP  Mean (SD) | Routine care  Mean (SD) | Unadjusted β (95%CI)^1^ | Adjusted β (95%CI)^2^ |
| --- | --- | --- | --- | --- | --- |
|  | (n=451) | (n=252) | (n=199) |  |  |
| Imputed GWG at delivery3 | 8.31 (0.58) | 8.29 (0.58) | 8.33 (0.58) | -0.037 (-0.232, 0.158) | -0.000 (-0.002, 0.002) |
| GWG rate (kg/week) | 0.28 (0.01) | 0.28 (0.01) | 0.28 (0.01) | 0.001(-0.002, 0.004) | -0.000 (-0.001, 0.001) |
| GWG Adequacy (%)^4^ | 60.34 (1.90) | 60.32 (1.89) | 60.38 (1.90) | -0.064 (-0.482, 0.355) | 0.050 (-0.242, 0.342) |

ENP, Enhanced Nutrition; GWG, gestational weight gain

^1^ Cluster analysis based on 12 HC was used to derive β (95%CI)

^2^ Accounted for maternal age, weight, BMI, education, occupation, GA weeks at enrollment, and duration from enrollment and 3rd trimester (only for observed GWG), accounting for health center location

^3^ gestational weight at delivery was imputed by a mixed-effects model. We fitted a two-level linear mixed-effect model with maternal weight at delivery as the outcome. Fixed effects included a quadratic term of gestational age at each weight measurement, baseline weight, gestational age at enrollment, maternal age, body mass index, education, and occupation. Random intercepts were specified at the individual level with a random slope for gestational age. Fixed-effect predictions were computed at the gestation age of pregnancy outcome, where the centered gestational age term equals zero. These fixed effects were combined with the estimated random intercepts at the individual levels to generate personalized predictions of maternal weight at the gestational age of delivery

^4^ GWG Adequacy (%) = (Observed GWG ÷ Recommended GWG) × 100. Recommended GWG at the last observed weight measures = (BMI specific expected first trimester weight/13.86 weeks)*(13.86 weeks-GA at first observed or imputed weight measure)) + ((GA at last weight measure -13.86 wks)*BMI specific recommended mean rate of GWG in the second and third trimester. BMI specific expected first-trimester weight gain (2 kg for underweight or normal weight, 1 kg for overweight, 0.5 kg for women classified as obese) for the number of weeks since the observed or imputed first-trimester weight until the end of the first trimester (136/7 weeks=13.86 weeks). The weight gain up to the last weight measure based on the BMI specific recommended mean rate of weight gain in the second and third trimesters (0.51 kg/week for underweight, 0.42 kg/week for normal weight, 0.28 kg/week for overweight, and 0.22 kg/week for obesity)

**Supplementary Table 5. ENP Package Effect by Subgroups**

| Outcomes | Total  Mean (SD) | ENP  Mean (SD) | Routine care Mean (SD) | Unadjusted β^1^ (95%CI) | Adjusted^2^ β  (95%CI)^2^ |
| --- | --- | --- | --- | --- | --- |
| **A. Total ENAT Cohort** | n=1806 | n=1006 | n=800 |  |  |
| **Subgroups** |  |  |  |  |  |
| ***B. MUAC >23 cm*** | n=1145 | n=632 | n=513 |  |  |
| GWG rate (kg/week) | 0.291 (0.161) | 0.280 (0.141) | 0.305 (0.182) | -0.015 (-0.059, 0.029) | -0.013 (-0.053, 0.028) |
| Observed GWG (kg) | 5.496 (3.301) | 5.405 (3.069) | 5.609 (3.566) | 0.126 (-0.879, 1.132) | -0.087 (-0.887, 0.715) |
| ***C.MUAC≤23 cm**** | n=644 | n=374 | n=270 |  |  |
| GWG rate (kg/week) | 0.296 (0.147) | 0.294 (0.142) | 0.297 (0.153) | 0.008 (-0.029, 0.046) | 0.006 (-0.027, 0.039) |
| Observed GWG (kg) | 5.651 (3.126) | 5.708 (3157) | 5.572 (3.087) | 0.302 (-0.403, 1.007) | 0.201 (-0.443, 0.845) |
| ***D. BMI<18.5 at enrollment*** | n=305 | n=198 | n=107 |  |  |
| GWG rate (kg/week) | 0.319 (0.145) | 0.311 (0.147) | 0.333 (0.140) | -0.012 (-0.070, 0.046) | -0.011 (-0.059, 0.038) |
| Observed GWG (kg) | 6.611 (3.301) | 6.432 (3.329) | 6.943 (3.238) | -0.237 (-1.640, 1.166) | -0.103 (-1.109, 0.903) |
| ***E. BMI≥18.5 at enrollment*** | N=1500 | N=807 | N=693 |  |  |
| GWG rate (kg/week) | 0.287 (0.158) | 0.279 (0.140) | 0.297 (0.176) | -0.009 (-0.051, 0.032) | -0.006 (-0.045, 0.033) |
| Observed GWG (kg) | 5.326 (3.189) | 5.295 (3.007) | 5.362 (3.189) | 0.174 (-0.706, 1.054) | 0.012 (-0.727, 0.751) |

ENAT, Enhancing Nutrition and Antenatal Infection Treatment; ENP, Enhanced Nutrition Package; BEP, Balanced energy-protein; GWG, gestational weight gain; MUAC, mid-upper arm circumference; BMI, body mass index

^1^ Cluster analysis based on 12 HC was used to derive β (95%CI)

^2^ Accounted for maternal age, weight, BMI, education, occupation, GA wks at enrollment, and duration from enrollment and 3rd trimester (only for observed GWG), accounting for health center location

* MUAC <23 cm group was targeted with Balanced Energy Protein Supplementation in the Enhanced Nutrition Package (ENP) Arm
